# Mfn2 Overexpression Attenuates MPTP Neurotoxicity In Vivo

**DOI:** 10.3390/ijms22020601

**Published:** 2021-01-09

**Authors:** Fanpeng Zhao, Quillan Austria, Wenzhang Wang, Xiongwei Zhu

**Affiliations:** Department of Pathology, Case Western Reserve University, Cleveland, OH 44106, USA; fxz111@case.edu (F.Z.); qaustria11@gmail.com (Q.A.); wxw157@case.edu (W.W.)

**Keywords:** Parkinson’s disease, Mfn2, mitochondrial dynamics, MPTP, neurodegeneration

## Abstract

Mitochondrial dysfunction represents a critical event in the pathogenesis of Parkinson’s disease (PD). Increasing evidence demonstrates that disturbed mitochondrial dynamics and quality control play an important role in mitochondrial dysfunction in PD. Our previous study demonstrated that MPP^+^ induces mitochondrial fragmentation in vitro. In this study, we aimed to assess whether blocking MPTP-induced mitochondrial fragmentation by overexpressing Mfn2 affords neuroprotection in vivo. We found that the significant loss of dopaminergic neurons in the substantia nigra (SN) induced by MPTP treatment, as seen in wild-type littermate control mice, was almost completely blocked in mice overexpressing Mfn2 (hMfn2 mice). The dramatic reduction in dopamine neuronal fibers and dopamine levels in the striatum caused by MPTP administration was also partially inhibited in hMfn2 mice. MPTP-induced oxidative stress and inflammatory response in the SN and striatum were significantly alleviated in hMfn2 mice. The impairment of motor function caused by MPTP was also blocked in hMfn2 mice. Overall, our work demonstrates that restoration of mitochondrial dynamics by Mfn2 overexpression protects against neuronal toxicity in an MPTP-based PD mouse model, which supports the modulation of mitochondrial dynamics as a potential therapeutic target for PD treatment.

## 1. Introduction

Parkinson’s disease (PD) is a progressive neurological disorder that results from the selective loss of dopaminergic (DA) neurons in the substantia nigra (SN) pars compacta region of the midbrain [1]. The characteristic motor impairments of PD include resting tremors, bradykinesia, rigidity, and postural instability [2]. Although genetic research on PD has led to the identification of several monogenic forms of the disorder and numerous genetic risk factors increasing the risk of developing PD, only 5–10% of patients suffer from the monogenic form of PD, which can be caused by autosomal-dominant mutations in SNCA, LRRK2, and VPS35 and autosomal-recessive mutations in PINK1, DJ-1, and Parkin [3,4]. The majority of PD cases are idiopathic, in which environmental factors such as exposure to environmental neurotoxins play an important role [5]. Rodents or nonhuman primates exposed to various neurotoxins, especially 1-methyl-4-phenyl-1,2,3,6-tetrahydropyridine (MPTP) [6], remain the most widely used Parkinson disease models. MPTP produces reliable lesions of the nigrostriatal dopaminergic pathway and causes dopaminergic neuron loss and motor behavioral deficits after systemic administration in rodents [7,8]. MPTP-based mouse models are used to understand the mechanisms underlying the demise of dopaminergic neurons in PD and to test symptomatic and neuroprotective drugs [8].

Mitochondrial dysfunction represents a critical step in the pathogenesis of PD [9,10,11,12] because (1) mitochondrial complex I deficiency is a consistent feature of PD, (2) the products of many PD-associated genes locate to mitochondria and impact mitochondrial function, and (3) many neurotoxins causing parkinsonism in rodents, nonhuman primates, and humans are specific inhibitors of mitochondrial function. Mitochondria are highly dynamic organelles with the ability to continuously fuse and divide to maintain their shape, number, and proper distribution [13], and increasing evidence suggests a critical role of abnormal mitochondrial dynamics in mitochondrial dysfunction and neuronal dysfunction in various neurodegenerative diseases, including PD [11,14,15]. Mitochondrial dynamics are regulated by several large GTPases, including optic atrophy 1 (OPA1), mitofusin 1 (Mfn1), and mitofusin 2 (Mfn2) on the fusion side and dynamin-like protein 1 (DLP1), mitochondrial fission 1 protein (Fis1), and mitochondrial fission factor (Mff) on the fission side [16]. Studies by multiple groups have demonstrated that proteins encoded by familial PD-associated genes are involved in the regulation of mitochondrial dynamics and homeostasis [17,18,19,20,21]. For example, LRRK2 and VPS35 interact with DLP1, and PD-associated LRRK2 or VPS35 mutations cause DLP1-dependent mitochondrial fragmentation. PD-associated PINK1 and Parkin mutations likely tip the mitochondrial fission/fusion balance through enhanced degradation of Mfn2 and cause excessive mitochondrial fission. PD-associated neurotoxins also cause mitochondrial fragmentation, which contributes to their toxic effects on mitochondrial function and cell death. For example, paraquat, the neurotoxin associated with a twofold increased risk of PD, induces significantly increased DLP1 expression and mitochondrial fragmentation both in vitro and in vivo [22].

We previously demonstrated that MPP^+^, a toxic metabolite of MPTP, induces a biphasic increase of DLP1-dependent mitochondrial fragmentation in both SH-SY5Y neuroblastoma cells and primary dopaminergic midbrain neurons in vitro [23]. Inhibition of mitochondrial fission by DLP1 knockdown alleviates MPP^+^-induced mitochondrial bioenergetics deficits and cell death, which suggests a critical role of mitochondrial dynamic changes in mediating the toxic effects of MPP^+^, in addition to its direct inhibitory effect on complex I of the electron transport chain [23]. In the current study, we aimed to determine whether restoration of mitochondrial dynamics by increasing mitochondrial fusion through Mfn2 overexpression rescues MPTP-induced deficits in vivo.

## 2. Results

### 2.1. Mfn2 Overexpression (OE) Reduces MPTP-Induced Motor Deficits

**Figure 1:**
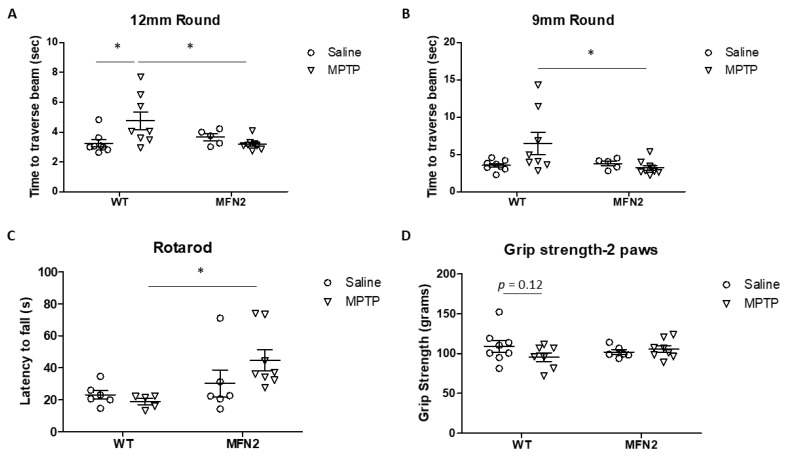
Mfn2 overexpression rescued MPTP-induced motor deficits. MFN2 Tg mice and WT mice were treated with MPTP (20 mg/kg) or vehicle control saline once a day for five consecutive days. Motor function was measured by the beam walking test (**A**,**B**) and the rotarod test (**C**) on days 6–7 after the last injection. The grip strength of two paws (**D**) was measured on day 7 before sacrifice (*n* = 5–8/group; * *p* < 0.05 compared to the control).

We developed and characterized a transgenic mouse model with enhanced mitochondrial fusion by overexpressing human Mfn2 under the Thy1.2 promoter (hMFN2 Tg mouse) in a prior study where hMFN2 Tg mice demonstrated elongated mitochondria and rescued paraquat-induced mitochondrial fragmentation in vivo [22]. To determine whether and how the rescue of MPTP-induced mitochondrial fragmentation affects PD-related deficits in vivo, hMFN2 Tg mice and WT littermate control mice were subjected to a subchronic MPTP treatment scheme, which involved one intraperitoneal injection of 20 mg/kg of free-base MPTP daily for five consecutive days [8]. The motor function of MPTP- or saline-treated mice was measured on days 6–7 after the last injection. In the beam walking test, the walking time to traverse a 12 mm round beam (Figure 1A) and a 9 mm round beam (Figure 1B) was significantly longer in MPTP-treated WT mice compared to saline-treated WT mice. However, no changes in the walking time to traverse the beams were found in MPTP-treated hMFN2 Tg mice compared to saline-treated hMFN2 Tg or WT mice. In the rotarod test, the average latency on the rotated rod decreased after MPTP treatment in WT mice but not in hMFN2 Tg mice (Figure 1C). The decreased two-paw grip strength in MPTP-treated WT mice was also restored in hMFN2 Tg mice (Figure 1D). These data suggested that Mfn2 OE protects mice from MPTP-induced motor function deficits.

### 2.2. Mfn2 OE Protects DA Neurons from MPTP-Induced Degeneration in the SN

**Figure 2:**
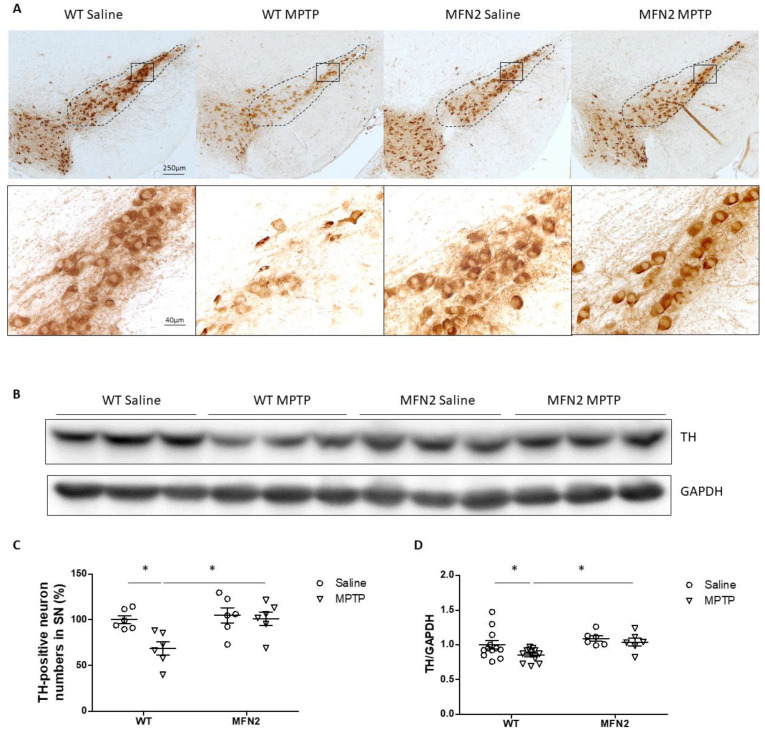
Mfn2 overexpression protected DA neurons from MPTP-induced degeneration in the SN. Seven days after the last injection, the mice were killed and half brains were fixed for tyrosine hydroxylase (TH) immunostaining. Representative pictures of dopaminergic neurons (**A**) in the SNpc area (dashed line) and quantification of relative neuronal numbers in the SN (**C**) are shown. The ventral midbrain was isolated from the other half brains and used for immunoblots (**B**). Quantification of relative TH protein levels in the ventral midbrain is shown (**D**). (*n* = 5–6/group; * *p* < 0.05 compared to the control).

DA neurons in the SN were examined by immunostaining for tyrosine hydroxylase (TH) in MPTP-treated mice after behavioral tests. As expected, MPTP administration led to the obvious loss of DA neurons in the SN in WT mice. However, little neuronal loss was noted in hMFN2 Tg mice after MPTP treatment (Figure 2A). Quantification analysis of the TH+-neuron number in the SN showed that MPTP caused about 30% significant DA neuronal loss in WT mice, which was almost completely blocked in hMFN2 Tg mice (Figure 2C). The Western blot experiment showed that MPTP administration resulted in significantly reduced TH protein levels in the ventral midbrain in WT mice, which was almost completely prevented in MFN2 Tg mice (Figure 2B,D). These data demonstrated the neuroprotective effects of Mfn2 OE against MPTP-induced nigral neuronal death in vivo.

### 2.3. MFN2 OE Rescues MPTP-Induced Oxidative Stress in the SN

**Figure 3:**
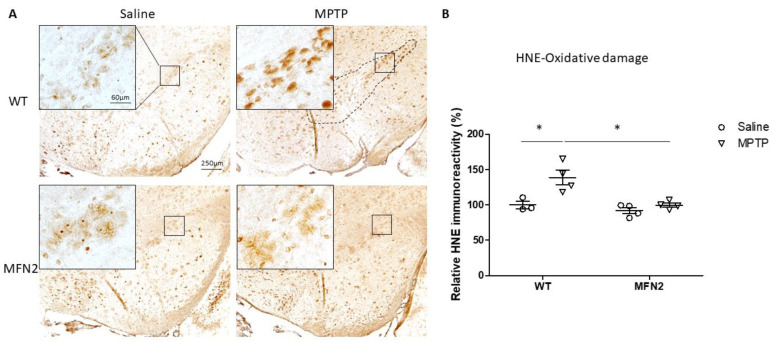
Mfn2 overexpression alleviated MPTP-induced oxidative stress in the SN. Fixed half brains of MPTP- or saline-treated mice were prepared, as shown in Figure 2A, and immunostained for 4-HNE. Representative pictures (**A**) and quantification (**B**) of relative 4-HNE immunoreactivity in the SN (dashed line) are shown. (*n* = 4; * *p* < 0.05 compared to the control).

An earlier study showed increased oxidative stress in DA neurons in PD, which may contribute to their final demise [24]. In this study, MPTP administration significantly elevated the immunoreactivity of 4-hydroxy-2-nonenal (4-HNE), an oxidative stress marker for lipid peroxidation, in neurons in the SN of WT mice by about 40% (Figure 3A,B). In contrast, no change in oxidative stress was observed in MPTP-treated hMFN2 Tg mice (Figure 3A,B), suggesting inhibition of MPTP-induced oxidative by Mfn2 OE in vivo.

### 2.4. Mfn2 OE Inhibits MPTP-Induced Activation of Microglia and Astrocytes in SN

**Figure 4:**
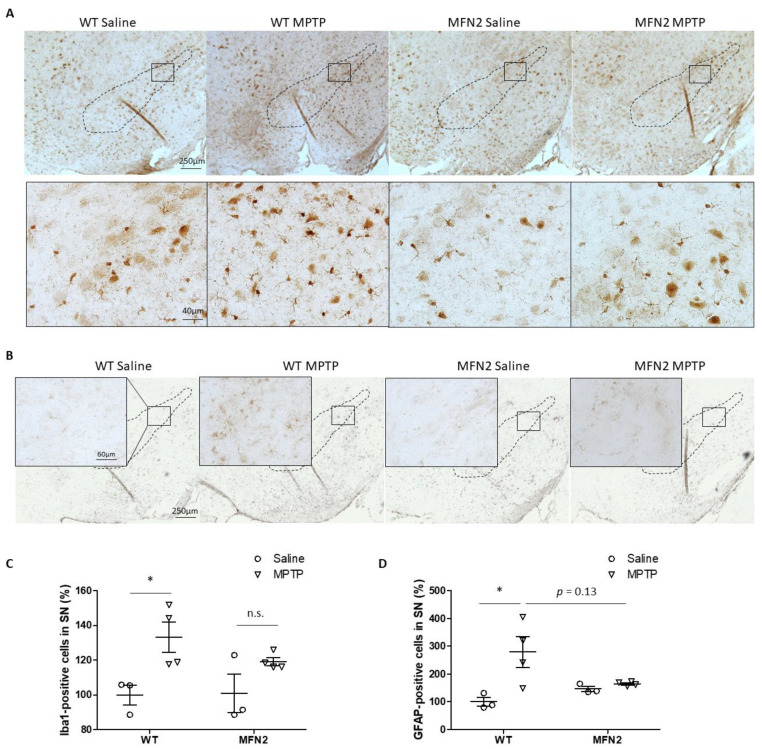
Mfn2 overexpression inhibited MPTP-induced astrocytosis and microgliosis in the SN. Fixed brains of MPTP- or saline-treated mice were immunostained for iba1 and GFAP. Representative pictures of microglia (**A**) and astrocytes (**B**) are shown. Quantification of the relative number of microglia (**C**) and astrocytes (**D**) in the SN (dashed line) is shown (*n* = 4; * *p* < 0.05 compared to the control. n.s., not significant).

The inflammatory response plays an important role in MPTP-induced neurotoxicity, and it is suggested as a promising interventional target for PD [25]. In this study, microgliosis and astrocytosis were investigated by immunostaining iba1 and glial fibrillary acidic protein (GFAP) in the SN in WT and hMFN2 Tg mice. In the saline-treated groups, there was no difference in the numbers of iba1-positive microglia and GFAP-positive astrocytes between WT and hMFN2 Tg mice at the basal level (Figure 4A,B). MPTP administration resulted in a significant increase in the number of iba1-positive microglia in the SN in WT mice but not in MFN2 Tg mice (Figure 4C). Robust activation of astrocytes in the SN was also found in WT mice after MPTP treatment but not in MPTP-treated MFN2 Tg mice (Figure 4D). Our data indicated that Mfn2 OE inhibits MPTP-induced activation of microglia and astrocytes in the SN and thus alleviates inflammatory response-involved neurotoxicity.

### 2.5. Mfn2 OE Alleviates MPTP-Caused Damage in the Striatum

**Figure 5:**
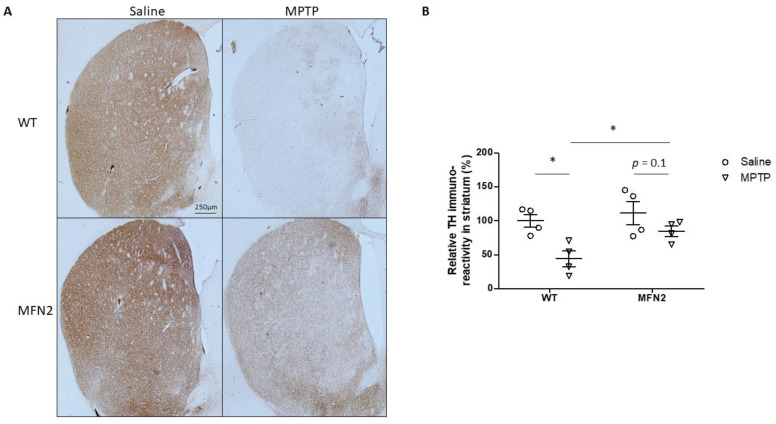
MFN2 overexpression rescued MPTP-induced loss of TH+-terminals in the striatum. Representative pictures of TH+ terminal fibers of dopaminergic neurons in the striatum (**A**) and quantification of the relative fiber density in the striatum (**B**) are shown. (*n* = 4; * *p* < 0.05 compared to the control).

Striatal dopaminergic terminal loss is an early and dominant feature of PD, and degeneration of the nigrostriatal system leads to symptomatic motor deficits [26]. MPTP treatment caused a dramatic decrease in the TH-positive fibers of the dorsolateral striatum in WT mice, but they were alleviated in hMFN2 Tg mice (Figure 5A). Quantification of TH immunoreactivity showed that MPTP caused less reduction of TH immunoreactivity in the striatum in MPTP-MFN2 mice (~30%) compared to MPTP-WT mice (~60%) (Figure 5B), which suggests that Mfn2 OE inhibits MPTP-induced degeneration of dopaminergic axons in the PD model.

### 2.6. Mfn2 OE Rescues MPTP-Induced Striatal Dopamine Loss

**Figure 6:**
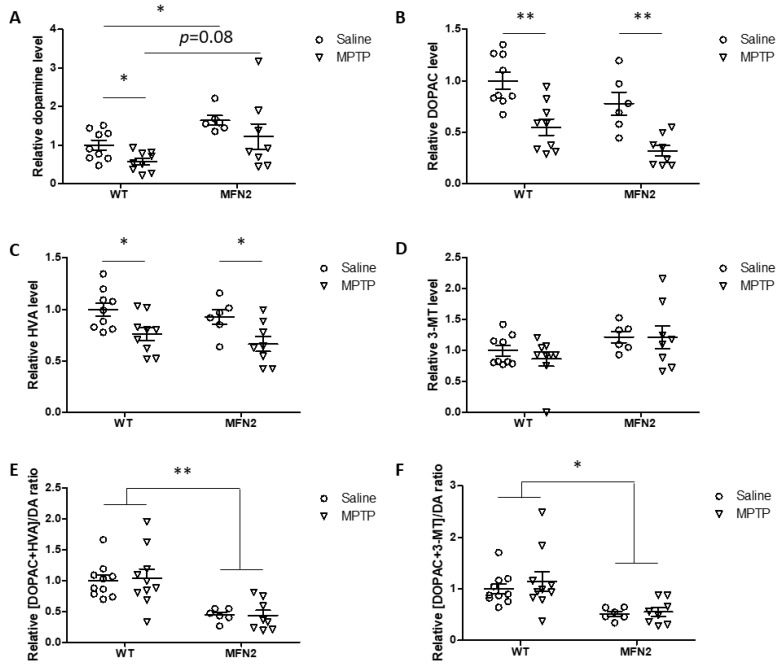
Mfn2 overexpression rescued MPTP-induced striatal dopamine loss. Total dopamine (**A**), DOPAC (**B**), HVA (**C**), and 3-MT (**D**) in striatal tissues from MPTP-treated mice measured by HPLC. Dopamine turnover was calculated as [DOPAC+HVA]/DA (**E**) and [DOPAC+3-MT]/DA (**F**). All the data are shown as relative values. The average levels in the saline-treated WT group were normalized to 1 (*n* = 6–10; * *p* < 0.05 and ** *p* < 0.01 compared to the control).

Striatal dopamine and its metabolites were measured by high-performance liquid chromatography (HPLC). Our results showed that the basal levels of total striatal dopamine were elevated in hMFN2 Tg mice compared with those in WT mice in saline-treated controls (Figure 6A). Consistently, dopamine turnover significantly decreased in hMFN2 Tg mice compared to WT mice at the basal level (Figure 6E,F), suggesting that MFN2 likely affects the metabolism of dopamine in vivo. MPTP treatment caused a significant decrease in striatal dopamine levels by around 50% in WT mice compared to saline-treated WT mice. While MPTP also resulted in a trend toward reduction in dopamine levels, which did not reach significance, in the striatum of hMFN2 Tg mice compared to saline-treated hMFN2 Tg mice (Figure 6A), dopamine levels in MPTP-treated hMFN2 Tg mice remained comparable to those in saline-treated WT mice. Dopamine metabolites 3,4-dihydroxyphenylacetic acid (DOPAC) and homovanillic acid (HVA), but not 3-methoxytyramine (3-MT), significantly decreased with MPTP exposure in both WT and hMFN2 Tg mice (Figure 6B–D). Dopamine turnover slightly increased in MPTP-treated WT mice but was not at all affected by MPTP in hMFN2 Tg mice compared to saline-treated controls (Figure 6E,F). More detailed analysis revealed a trend toward increased dopamine turnover in MPTP-treated WT male mice but not in female WT mice (data not shown), consistent with prior studies demonstrating that female C57Bl/6 mice are significantly less sensitive to MPTP-induced neurotoxicity compared to male mice [27,28,29]. However, this trend was completely prevented in MPTP-treated male hMFN2 Tg mice (data not shown).

### 2.7. Mfn2 OE Inhibits MPTP-Induced Inflammation in the Striatum

**Figure 7:**
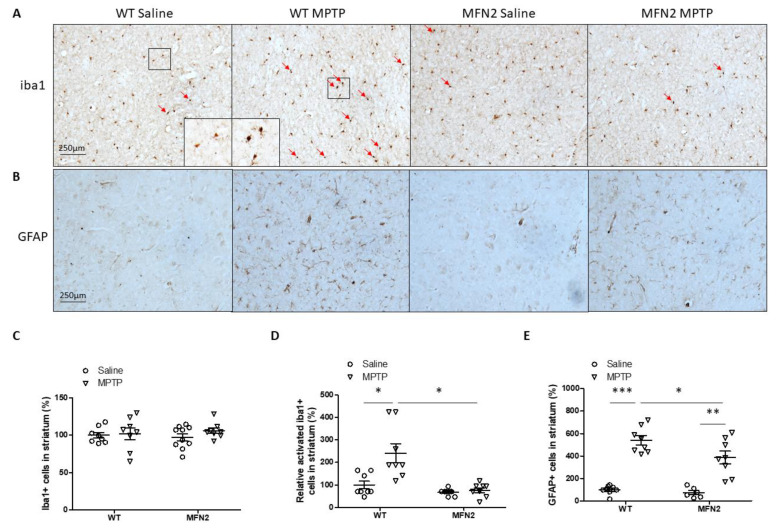
Mfn2 overexpression inhibited MPTP-induced inflammation in the striatum. Representative pictures of iba1 (**A**) staining in the striatum and quantification of total (**C**) or relative activated (**D**) microglia in the striatum are shown (Red arrows indicate activated microglia). Representative pictures of GFAP staining in the striatum (**B**) and quantification of astrocytes (**E**) in the striatum are shown (*n* = 4; * *p* < 0.05, ** *p* < 0.01, and *** *p* < 0.001 compared to the control).

In our subchronic MPTP mouse model, the number of iba1-positive microglia in the striatum did not change 7 days after the last MPTP injection in both WT and hMFN2 Tg mice (Figure 7A). However, morphologic analysis showed that the number of activated microglia in the striatum significantly increased in WT mice but not in hMFN2 Tg mice (Figure 7A,C,D). The number of astrocytes in the striatum significantly increased after MPTP treatment in both WT and hMFN2 Tg mice (Figure 7B), but Mfn2 OE significantly suppressed MPTP-induced activation of astrocytes in the striatum (Figure 7E). These data suggested that MPTP-induced microgliosis and astrogliosis in the striatum are inhibited in hMFN2 Tg mice.

## 3. Discussion

Using a subchronic MPTP-based mouse model of PD, this study provides evidence that restoration of mitochondrial dynamics by overexpressing Mfn2 effectively reduces MPTP-induced PD-related motor deficits and brain pathology. This conclusion is deduced from the following observations: First, MPTP-induced motor deficits and DA neuronal death in WT mice are rescued in hMFN2 Tg mice. Second, Mfn2 OE blocks oxidative damage, microglial activation, and reactive astrogliosis caused by MPTP in the nigrostriatal pathway in vivo. Third, MPTP-induced loss of striatal DA neuronal axons and reduction in striatal dopamine levels in WT mice are inhibited by Mfn2 OE in vivo. Our findings suggest that restoration of mitochondrial dynamics could be a promising therapeutic target for PD.

In our previous study, we observed increased DLP1 levels and a trend toward decreased Mfn1 and Mfn2 levels in the midbrain of sporadic PD patients [22], implying that an imbalance in mitochondrial fission and fusion is likely involved in mitochondrial dysfunction and the pathogenesis of PD. PD-related neurotoxins, including MPP^+^ and paraquat, enhance the production of the mitochondrial fission protein DLP1 and cause mitochondrial fragmentation even in low concentrations that did not induce cell death [22,23]. However, some other groups found MPP^+^-induced reduction in the cristae structure and swelling instead of mitochondrial fragmentation [30]. Notably, a higher concentration of MPP^+^ was used in the latter study, which could cause acute cell death that manifests as mitochondrial swelling, masking the specific effects on mitochondrial fragmentation as an early step during MPP^+^-induced mitochondrial dysfunction [30]. Supporting the involvement of mitochondrial fragmentation in MPP^+^-induced mitochondrial dysfunction, several groups, including our own, have demonstrated that blocking DLP1 function by DLP1 knockdown or mitochondrial division inhibitors (i.e., mdivi-1 or p110 peptide) is sufficient to attenuate MPP^+^- or MPTP-induced mitochondrial dysfunction and neurotoxicity in PD cell culture and mouse models [23,31,32]. Nevertheless, DLP1 knockdown causes perinuclear accumulation of mitochondria and synaptic deficits [33], which suggests that normal DLP1 expression and mitochondrial fission are critical to ensure the proper motility and distribution of mitochondria important for physiological mitochondrial and neuronal function [11]. It therefore appears that enhancing mitochondrial fusion events could be a safer method to restore mitochondrial dynamics than inhibiting fission events as a therapeutic strategy for the treatment of PD. In this study, the first question we addressed is whether genetic Mfn2 OE improves motor behaviors in a subchronic MPTP-based mouse model. We found that Mfn2 OE provides neuroprotection against MPTP-induced motor deficits. Consistent with the MPTP-induced neurotoxicity in motor functions, parkinsonism-related neurotoxicity of SN DA neurons and striatal terminals were also inhibited by MFN2 OE in vivo.

One interesting finding of our study is that Mfn2 overexpression provides almost complete protection against MPTP-induced loss of TH+-DA neurons in the SN but relatively less protection against MPTP-induced loss of TH+-dopaminergic terminals in the striatum. A similar pattern of protection by mdivi-1 or OPA1 overexpression was also reported in other studies in an MPTP model of PD [30,32]. TH+ staining in the striatum marks the axon terminal derived from the SN. It has been consistently shown that MPTP causes earlier and more severe damage to axonal terminals in the striatum than cell bodies in the SN, supporting a dying-back mode of degeneration in PD [26]. The relative ineffectiveness of Mfn2 overexpression in the protection of MPTP-induced dopaminergic terminals suggests that other factors, in addition to mitochondrial fragmentation, must be involved in mediating the initiation of damage to axonal terminals. In this regard, Mfn2 overexpression only leads to partial protection against MPTP-induced GFAP activation in the striatum, suggesting that increased astrocytosis might be involved. The full protection of DA neurons in the SN of hMFN2 Tg mice supported a critical role of abnormal mitochondrial dynamics in mediating the cellular demise of the cell bodies of dopaminergic neurons.

Another important finding of the current study is that Mfn2 likely regulates the metabolism of dopamine in vivo. Our data showed that basal dopamine levels in Mfn2 Tg mice were significantly elevated compared to WT mice. In this regard, the relative ratio of dopamine metabolites to dopamine was also significantly reduced in hMFN2 OE mice, suggesting that Mfn2 OE or changes in mitochondrial morphology lead to higher dopamine levels by inhibiting dopamine biodegradation in vivo. A previous study showed a severe reduction in dopamine levels and increased dopamine turnover in the striatum of conditional Mfn2-knockout mice [34], suggesting a specific role of Mfn2 or mitochondrial dynamics in dopamine metabolism at the basal level, which will be pursued further. As MPTP caused a decrease in striatal dopamine levels at similar magnitudes in hMFN2 Tg mice as in WT mice and that inhibition of mitochondrial fission by mdivi-1 or expression of the dominant negative DLP1 mutant slightly rescued MPTP-induced reduction in striatal dopamine [32], it is less likely that the rescue of mitochondrial morphology plays a major role in the rescue of MPTP-induced striatal dopamine levels.

Taken together, our study demonstrates that overexpression of Mfn2 provides protection against MPTP-induced motor deficits, loss of dopaminergic neurons in the SN, and loss of dopaminergic axons in the striatum. Mfn2 overexpression provides an extra benefit to boost dopamine levels in the striatum. Our findings suggest that enhancing mitochondrial fusion by Mfn2 OE may be a potential therapeutic strategy to reduce PD pathologies.

## 4. Materials and Methods

### 4.1. Animals and Treatment

Animal studies were approved by the Institutional Animal Care and Use Committee (IACUC) of Case Western Reserve University (protocol#2016-0050; approval date: 06/18/2019). All the mice in this study were maintained under constant environmental conditions in the Animal Research Center of Case Western Reserve University, with free access to food and water. The development of Mfn2 transgenic (Tg) mice appeared normal compared to non-Tg littermates. Female and male 3-month-old WT and Tg mice were used in the experiments. MPTP administered to the animals was dissolved in 0.9% sterile saline and injected intraperitoneally. Tg mice and WT littermate controls received either a dose of 20 mg/kg of free-base MPTP or an equal volume of a vehicle once a day for 5 consecutive days, and 6–7 days after the final injection, the motor function of these mice was tested and then the mice were sacrificed for tissue collection. Half brains were fixed overnight at 4 °C in 10% neutral buffered formalin and embedded in paraffin for immunohistochemistry. Striatal tissues were dissected from the other half brains for HPLC analysis.

### 4.2. Measurement of Motor Function

The motor function of the mice was tested 6–7 days after the last MPTP injection. In the rotarod test, a mouse rotarod apparatus (Harvard apparatus, Holliston, MA, USALE8200) was used to measure motor coordination in treated mice. The mice were trained to stay on the rotarod for 3 min at constant speeds (4, 10, and 20 rpm) for 3 consecutive days just before the test. Six days after the last MPTP administration, the latency to fall in the rotarod test was measured at a speed accelerating from 4 to 40 rpm in 2 min. The mice were tested 3 times with a rest of 5 min between each trial. In the beam walking test, the walking time to traverse narrow beams was recorded. Each mouse crossed a 12 mm round beam 3 times and then a 9 mm round beam 3 times. The muscular strength of each mouse was measured by a grip strength test meter (Bioseb, Pinellas Park, FL, USA, BIO-GS3). Two forepaws of each mouse were placed on a bar that was connected to the machine, and the mouse’s tail was pulled back. Each mouse was tested 3 times to measure muscular strength. The recorded values in all tests were averaged for each mouse and then used for statistical analysis.

### 4.3. Antibodies and Chemicals

The primary antibodies used in this study included mouse anti-TH (Millipore, Temecula, CA, USA, MAB318), rabbit anti-GAPDH (Cell Signaling, Danvers, MA, USA, 2118), rabbit anti-4-HNE (Alpha Diagnostics, San Antonio, TX, USA, HNE11-S), GFAP (Invitrogen, Rockford, IL, USA, PA5-16291), and iba1 (Wako, Osaka, Japan, 019-19741). Secondary antibodies used in this study included anti-mouse/rabbit HRP-linked secondary antibody (Cell Signaling, Danvers, MA, USA, 7076 and 7074) and goat anti-mouse or goat anti-rabbit (Millipore, Temecula, CA, USA, AP124 or AP132) peroxidase-conjugated antibody. 1-Methyl-4-phenyl-1,2,3,6-tetrahydropyridine (MPTP) hydrochloride (Santa Cruz, CA, USA, 23007-85-4) was purchased and dissolved in saline for mice treatment.

### 4.4. Immunohistochemistry

Mouse brains were fixed overnight at 4 °C in 10% neutral buffered formalin. Then, paraffin-embedded brains were sliced into consecutive coronal sections of 14 μm for the midbrain and striatum. The sections were sequentially incubated overnight at 4 °C with appropriate primary antibodies. The sections were then incubated with either goat anti-mouse or goat anti-rabbit peroxidase-conjugated antibody, followed by the species-specific peroxidase–antiperoxidase complex (Jackson, West Grove, PA, USA, 223005024 or 323005024). 3-3′-Diaminobenzidine (Dako, Carpinteria, CA, USA, K3468) was used as a chromogen. For quantification of dopaminergic neurons, multiple sections of the SN region were immunostained for each mouse using tyrosine hydrolase (TH) antibody. The number of dopamine neurons in the SNpc was estimated by counting the TH-positive neurons of five coronal sections per mouse, which were distributed about every 100 μm along the rostral–caudate axis of the SN (−3.08 to −3.64 mm caudal to bregma) [35]. Densitometric analysis of the TH-immunostained striatum area was performed using ImageJ 1.44 (https://imagej.nih.gov/ij/download.html) software on mouse sections.

### 4.5. Western Blot Analysis

Mouse brain tissues were carefully dissected and homogenized with RIPA lysis buffer plus a protease inhibitor mixture (Roche, Penzberg, Germany, 5892791001/4906837001). Homogenates were centrifuged at 14,000 rpm for 20 min and the supernatants collected and protein levels determined using the BCA assay (Thermo Fisher Scientific, Waltham, MA, USA, 23225). Equal amounts of total protein extracts were resolved by SDS-PAGE and transferred to Immobilon-P (Millipore, Temecula, CA, USA, IPVH00010). After blocking with 10% nonfat milk, appropriate primary and secondary antibodies were applied, and blots were developed with an Immobilon Western Chemiluminescent HRP substrate (Millipore, Temecula, CA, USA, WBKLS0500). 

### 4.6. HPLC Measurements of Striatal Dopamine and Metabolites

Microdissected striatal tissues were frozen on dry ice and then stored at −80 °C until analysis for dopamine, DOPAC, 3-MT, and HVA contents using HPLC. Dopamine and metabolite concentrations were analyzed at the Vanderbilt University Neurochemistry Core via high-performance liquid chromatography (HPLC), as described previously [36].

### 4.7. Statistical Analysis

All data are presented as mean ± standard error of means (SEMs). For analysis of statistical differences between three or more groups, one-way ANOVA with Bonferroni’s multiple comparison tests was applied. *p*-values are indicated by asterisks (*** *p* < 0.001; ** *p* < 0.01; * *p* < 0.05).

## Data Availability

The data presented in this study are available on request from the corresponding author.
